# Exosomes Derived From Umbilical Cord Mesenchymal Stem Cells Treat Cutaneous Nerve Damage and Promote Wound Healing

**DOI:** 10.3389/fncel.2022.913009

**Published:** 2022-06-30

**Authors:** Ziying Zhu, Xiaona Zhang, Haojie Hao, Heran Xu, Jun Shu, Qian Hou, Min Wang

**Affiliations:** ^1^College of Chemistry and Materials Engineering, Beijing Technology and Business University, Beijing, China; ^2^The First Medical Center, Chinese People’s Liberation Army General Hospital, Beijing, China; ^3^Medical Innovation Research Center, Chinese People’s Liberation Army General Hospital, Beijing, China

**Keywords:** wound repair, regeneration, exosome, umbilical cord mesenchymal stem cells, cutaneous nerve regeneration, nerve growth factor

## Abstract

Wound repair is a key step in the treatment of skin injury caused by burn, surgery, and trauma. Various stem cells have been proven to promote wound healing and skin regeneration as candidate seed cells. Therefore, exosomes derived from stem cells are emerging as a promising method for wound repair. However, the mechanism by which exosomes promote wound repair is still unclear. In this study, we reported that exosomes derived from umbilical cord mesenchymal stem cells (UC-MSCs) promote wound healing and skin regeneration by treating cutaneous nerve damage. The results revealed that UC-MSCs exosomes (UC-MSC-Exo) promote the growth and migration of dermal fibroblast cells. In *in vitro* culture, dermal fibroblasts could promote to nerve cells and secrete nerve growth factors when stimulated by exosomes. During the repair process UC-MSC-Exo accelerated the recruitment of fibroblasts at the site of trauma and significantly enhanced cutaneous nerve regeneration *in vivo*. Interestingly, it was found that UC-MSC-Exo could promote wound healing and skin regeneration by recruiting fibroblasts, stimulating them to secrete nerve growth factors (NGFs) and promoting skin nerve regeneration. Therefore, we concluded that UC-MSC-Exo promote cutaneous nerve repair, which may play an important role in wound repair and skin regeneration.

## Introduction

The skin is the largest organ of the human body and has an important role in resisting the invasion of external bacteria, regulating body temperature, sensation, and other aspects (Woodby et al., 2020). Skin trauma and its consequences are one of the major public health concerns worldwide and lead to substantial medical expenses every year (Sen et al., 2009). Wound repair is a complex and orderly biological process governed by multifaceted, multifactorial regulation, which is a key step in the treatment of skin injury caused by burns, surgery, and trauma. Although the skin tissue has a certain self-repair ability (Singer and Clark, 1999), but poor wound healing, in addition to affecting appearance, especially in the exposed site, not only leads to impaired skin function, such as altered perceptions of pain, temperature, and touch (Oualla-Bachiri et al., 2020; Torre et al., 2020) but also results in tissue infection and necrosis and even serious local or systemic dysfunction (Roh et al., 2017). Due to the loss or incomplete recovery of the subcutaneous nerves and nerve endings, the patient may exhibit symptoms such as hyperesthesia (including itching) and dysesthesia, leading to decreased sensory and motor function and ultimately affecting the patient’s quality of life.

Skin wound healing is the focus and hotspot of clinical research. With the development of molecular biology, the understanding of skin wound healing mechanisms has gradually deepened. Skin wound healing includes the hemostasis stage, inflammatory response, proliferation, and remodeling phases (Shaw and Martin, 2009; Gonzalez et al., 2016; Tottoli et al., 2020). Based on accumulating evidence, the migration and proliferation of keratinocytes, fibroblasts, endothelial cells, and leukocytes increase in the wound during wound repair, further promoting wound healing (Woodley, 2017; Dorschner et al., 2020; Piipponen and Li, 2020). The concomitant inflammatory response during skin wound healing stimulates the abundant sensory and motor nerves in the dermis and releases signals such as neuropeptides (Pradhan et al., 2009). Moreover, skin regeneration and scar healing both involve nerve repair. The growth of nerve axons is also affected by cells, extracellular matrix (ECM), and various regulatory factors in granulation tissue and scar tissue, and nerves and various regulatory healing factors form a complex network of interactions (Chéret et al., 2014). For example, during the wound healing process, cellular growth factors secreted by related cells, such as transforming growth factor-1 (TGF-β1), platelet-derived growth factors (PDGFs), vascular endothelial growth factors (VEGFs), epidermal growth factors (EGFs), fibroblast growth factors (FGFs), and insulin-like growth factors (IGFs) can promote cell growth and differentiation and have a decisive role in wound healing (Borena et al., 2015). The fact that impaired or delayed wound healing is classically observed in patients with diabetes, congenital, or other neuropathies suggests that nerve regeneration is a factor promoting skin wound healing (Falanga, 2005). Moreover, evidence indicates that cutaneous innervation is an important modulator of the normal wound healing process, and denervated skin could result in impaired wound healing (Smith and Liu, 2002; Buckley et al., 2012).

In recent years, many emerging skin regeneration techniques have been rapidly developed. Stem cells, biomaterial scaffolds, bioactive factors, etc. have been used to promote the regeneration of skin (Chen et al., 2020; Niimi et al., 2020; Tottoli et al., 2020). Extracellular vesicle-based therapeutics have shown promise in preclinical and clinical studies. Exosomes are cystic vesicles that are generated through the endosomal pathway and released outside the cell (Robbins and Morelli, 2014). These vesicles carry various biologically active substances, such as lipids, proteins, and nucleic acids, and play an important role in intercellular transport and information transmission (Aheget et al., 2020). Due to the regenerative ability and immunosuppressive properties of MSCs, they have been well used in clinical trials for the treatment of various diseases. At present, the study of stem cell exosomes mainly focuses on the regeneration and repair of multiple organs and systems, such as the cardiovascular system (Ibrahim et al., 2014), liver (Ying et al., 2017), and nerves (Ching and Kingham, 2015). Moreover, studies have shown that MSC-Exo are capable of acting on nearly all stages of wound healing, including controlling immune responses, inhibiting inflammation, promoting cell proliferation and angiogenesis, and reducing scarring during wound healing (Farinazzo et al., 2015; Su et al., 2017; Vesna et al., 2018; Dong et al., 2019; Vu et al., 2021). MSC-Exo participate in this process by activating multiple signaling pathways (Whyte et al., 2012; Wang X. et al., 2019; Yu et al., 2019), such as the PI3K/AKT pathway, Wnt/β-catenin signaling pathway, and Notch signaling pathway. Among them, the Wnt signaling pathway is involved in every process of wound healing from inflammation control to apoptosis after being activated by skin injury. Studies have shown that exosomes are involved in the regulation of Wnt signaling in wound healing (Houschyar et al., 2015; Zhang et al., 2015). Accumulating evidence has suggested that MSC-Exo treatment is emerging as a promising method of skin nerve repair and skin regeneration (Ferreira and Gomes, 2018; Wu et al., 2018; Casado-Díaz et al., 2020). Nevertheless, the underlying mechanism by which MSC-Exo promotes wound healing remains unclear.

Exosomes from adipose-derived stem cells (ADSC-Exo) can promote the proliferation of Schwann cells (SCs) through the regulation of related protein mRNA expression to promote nerve regeneration (Liu et al., 2020). Bone marrow stromal cell-derived exosomes (BMSC-Exo) can promote peripheral nerve regeneration through miRNA-mediated regeneration-related genes (Zhao et al., 2020). Umbilical cord mesenchymal stem cells (UC-MSCs) have the advantages of easy availability, greater proliferative capacity, and low immunogenicity (Wang et al., 2018). Studies have shown that UC-MSCs can differentiate into key cell types in the three germ layers, inhibit inflammation (Lee et al., 2017), and have the ability to repair tissue damage and modulate immune responses (Liu et al., 2018; Tsai et al., 2021). UC-MSCs have potential applications in regenerative medicine and have been demonstrated to repair tissue damage in many inflammatory and degenerative diseases (Shiue et al., 2019; Tsai et al., 2021). Notably, in the field of regenerative medicine, UC-MSC-Exo also show great therapeutic potential.

In this study, we explored the related mechanism by observing the effect of UC-MSC-Exo on the proliferation ability of fibroblasts and whether UC-MSC-Exo could improve the wound healing rate by promoting nerve repair. During wound healing, the MSC-Exo-treated wounds demonstrated a stronger ability to recruit fibroblasts, stimulate them to secrete neurotropic factors, and regenerate nerve fibers than the control-treated wounds. These results suggest that UC-MSC-Exo have promising functions in nerve regeneration in cutaneous wounds.

## Materials and Methods

### Animals and Ethical Approval

Six-week-old C57BL/6 male mice were purchased from the Beijing Vital River Laboratory Animal Technology Company. All animals were treated strictly in accordance with international ethical guidelines and the National Institutes of Health Guide concerning the Care and Use of Laboratory Animals. All animal experiments were carried out with the approval of the Animal Ethical and Welfare Committee (AEWC).

### Culture, Expansion, and Identification of Umbilical Cord Mesenchymal Stem Cells

Umbilical cord mesenchymal stem cells (UC-MSCs) were obtained from ScienCell (Carlsbad, CA, United States). Cells were plated at 5 × 10^6^ cells per 60 mm dish and were cultured in Dulbecco’s modified Eagle medium (DMEM, Invitrogen, United States) with 10% fetal bovine serum (FBS, Gibco, United States) and 1% penicillin-streptomycin (PS, Gibco, United States) and maintained at 37°C in a humidified atmosphere containing 5% CO_2_ with the medium changed every 24 h. The fifth-passage MSCs were cultured to approximately 90% and replaced with a serum-free medium (APPLIED CELL, China), and the medium was changed every 24 h. The cell culture supernatant was collected at each medium change and centrifuged, and the supernatant was collected to extract exosomes. For MSCs identification, when grown as adherent cultures in monolayers, cells were incubated with anti-CD79a-fluorescein isothiocyanate (FITC), anti-CD14-FITC, anti-CD45-FITC, anti-Thy1-FITC, CD73-PE, anti-CD105-PE (Biolegend, San Diego, CA, United States). A flow cytometric analysis was conducted on an FACS Calibur cytometer (BD FACS Calibur*™*, Becton-Dickinson, San Jose, CA, United States) and analyzed by using the Flowing Software analysis program.

### Isolation, Purification, and Identification of UC-MSC-Exo

Umbilical cord mesenchymal stem cells were grown to the logarithmic phase, at which time supernatants were collected, after which exosomes were collected by ultracentrifugation. The first step was performed at 2,000 × *g* for 30 min to eliminate cells and large cell debris. The following step was centrifugation again at 10,000 × *g* for 45 min and then passed through a 0.45 μm filter (Merck Millipore, Germany) to remove small cell debris. At each of these steps, the pellet was discarded, and the supernatant was used for the following steps.

For the purification of exosomes, the supernatant obtained above was then ultracentrifuged at 100,000 × *g* for 70 min at 4°C. Then, the supernatant was removed, and the pellet was washed in a large volume of phosphate-buffered saline (PBS, HyClone, United States) to eliminate contaminating proteins and centrifuged last time at the same high speed. After isolation of these exosomes, they were resuspended in PBS, and the protein content of the exosome suspension was determined using a BCA quantitation kit (Beyotime, Shanghai, China).

The morphology and marker (Bioss, CD63 and CD81) expression of MSC-Exo were analyzed by transmission electron microscopy (TEM) (JEOL JEM-F200 200 kV, Japan) and western blotting, respectively. The size and concentration of exosomes were determined through Nanosight Tracking Analysis (NTA) by utilizing a ZetaView PMX 110 system (Particle Metrix, Meerbusch, Germany).

### Human Dermal Fibroblasts Migration Assay

Human dermal fibroblasts (HDFs) were obtained from ScienCell (Carlsbad, CA, United States). For the cell migration assay, human dermal fibroblasts (HDFs) were seeded at a density of 3.5 × 10^4^ cells/well in a two-well Ibidi silicone culture insert (Ibidi, Martinsried, Germany). After sufficient time for cell attachment (>24 h), the silicone insert was carefully removed. After two PBS washes, MSC-Exo prepared with DMEM were added, and a blank group was treated with DMEM as a control. Cell migration in the scratched area was observed at 0, 12, and 24 h under an inverted microscope (Olympus, Japan). The migration distance was analyzed using the ImageJ software, and the average value of the cell migration rate was calculated.

### Immunofluorescence

Human dermal fibroblasts were cultured in a 24-well plate, then washed with PBS and fixed in 4% paraformaldehyde for 30 min, permeabilized with 0.1% Triton-X100 for 30 min, followed by blocking with 5% bovine serum albumin (BSA) for 30 min, incubated with vimentin (1:100, Abcam) primary antibodies overnight at 4°C, and followed by incubation with a FITC-conjugated goat anti-rabbit Alexa 488 (1:500, Thermo Scientific) or goat anti-rabbit Alexa 647 (1:500, Thermo Scientific) for 1 h at room temperature. Nuclei were counterstained with 4′,6-diamidino-2-phenylindole (DAPI) (DAPI) (1:1,000, Thermo Scientific). Images were taken using a confocal microscope (Nikon, Japan).

### Quantitative Real-Time PCR

Dorsal skin total RNA was isolated using a total RNA extraction reagent (TRIzol, Invitrogen, United States), RNA was reverse-transcribed using a ReverTra Ace qPCR RT Kit (TOYOBO, Japan), cDNA was synthesized, and real-time PCR was conducted. The primer sequences used in these experiments are listed in Table 1. The data from real-time PCR experiments were analyzed by the comparative CT method as described in the manufacturer’s manual. The expression of nerve growth factors was assayed, and all results were confirmed by repeating the experiment 3 times.

**TABLE 1:** Sequences of primers used for quantitative RT-PCR analysis.

Primer	Forward	Reverse
VIPR1	TCATCCGAATCCTGCTTCAGA	AGGCGAACATGATGTAGTGTACT
CALCB	CACCTGTGTGACTCATCGGC	GGGCACGAAGTTGCTCTTCA
TACR1	TTGGCGTAGTTGTCGCGTTG	CGCGAATTAACTACGCACGA
TAC2	AGGGAGGGAGGCTCAGTAAG	GGCGGCTGTAGAGTC
TAC4	GAAGACGCTGCATGTATTG	CATATGCCATGACACATGCAG

### Western Blot

Western blot analysis was performed as previously described. Equal amounts of cell protein were size fractionated by sodium dodecyl sulfate/polyacrylamide (SDS-PAGE) gel electrophoresis and transferred onto polyvinylidene difluoride membranes. After transfer, the membranes were blocked with 5% BCA in Tris-buffered saline with Tween-20 (TBST)(Beyotime, Shanghai, China) for 1 h at room temperature with gentle shaking and incubated overnight at 4°C the following primary antibodies: CD63 (Bioss, bs-1523R), CD81 (Bioss, bs-6934R). All primary antibodies were diluted 1:1000. After TBST washes, the blots were incubated in goat anti-rabbit IgG H&L/HRP antibody (Bioss, bs-0295G-HRP) for 1 h at room temperature. The membranes were then washed 3 times, and the signals were visualized with enhanced chemiluminescence reagents (BeyoECL Star, Beyotime, China).

### Wound Closure Assay

C57BL/6 mice were randomly divided into a model control group and a treatment group. Then, the mice were housed in cages and kept adaptively for 7 days. Preexperimental animals were fasted for 12 h, and after anesthesia, the dorsum was shaved and cleaned. A circular full-thickness skin defect wound, 1 cm in diameter, was created by using surgical scissors on the back, and the wound was deep in the subcutaneous surface, forming an animal model of mechanical injury. After modeling, the mice were kept in a single cage, and the day of model establishment was recorded as day 0. Then, the mice were randomly divided into the control and MSC-Exo groups. Control group: the wound was treated with 100 μl of PBS (*n* = 8), dripped in the wounds; MSC-Exo group: the wound was treated with 100 μl of PBS containing 100 μg exosomes added externally (*n* = 10). Wound healing was evaluated on the basis of gross observation at days 0, 3, 7, and 14. The wound healing rate was calculated as follows: (primary wound size—residual wound size)/original wound size × 100%.

### Histological and Immunofluorescence Analysis

The wounded skin was excised and fixed with 4% paraformaldehyde (Solarbio, Beijing, China) for 24 h to prevent cell autolysis after death. Then, the sections were hydrated with running water for a certain period of time, followed by gradual dehydration with alcohol at different concentrations, embedding in paraffin and cutting into 5 μm sections. The sections were stained with hematoxylin-eosin (HE), and the histological changes of the wounds were visualized under a microscope.

Skin tissues were sectioned into 5 μm sections as above. For immunofluorescence double staining, sections were incubated overnight at 4°C with a mix of primary anti-PGP9.5 (GB11159-1, 1:1,000, Servicebio) and anti-GAP43 (bs-0154R, 1:4,000, Bioss) antibodies. Sections were incubated in Cy3 conjugated Goat Anti-Rabbit IgG (H+L) (GB21303, 1: 300, Servicebio) or HRP conjugated Goat Anti-Rabbit IgG (H+L) (GB23303, 1:500, Servicebio) secondary antibody for 1 h at room temperature. Images were captured by laser scanning confocal microscope (Olympus, Japan), and the confocal software was used for acquisition of the data and merging of the digital images. Each antibody was validated separately prior to use in double immunofluorescence.

### Statistical Analysis

Statistical analysis was performed using GraphPad Prism 9 (GraphPad Software). All data were evaluated using analysis of variance (ANOVA) followed by Bonferroni *post-hoc* testing. Data are presented as the mean with a standard error of the mean (SEM), and *P* < 0.05 was considered statistically significant.

## Results

### Origin and Identification of Exosomes

**FIGURE 1:**
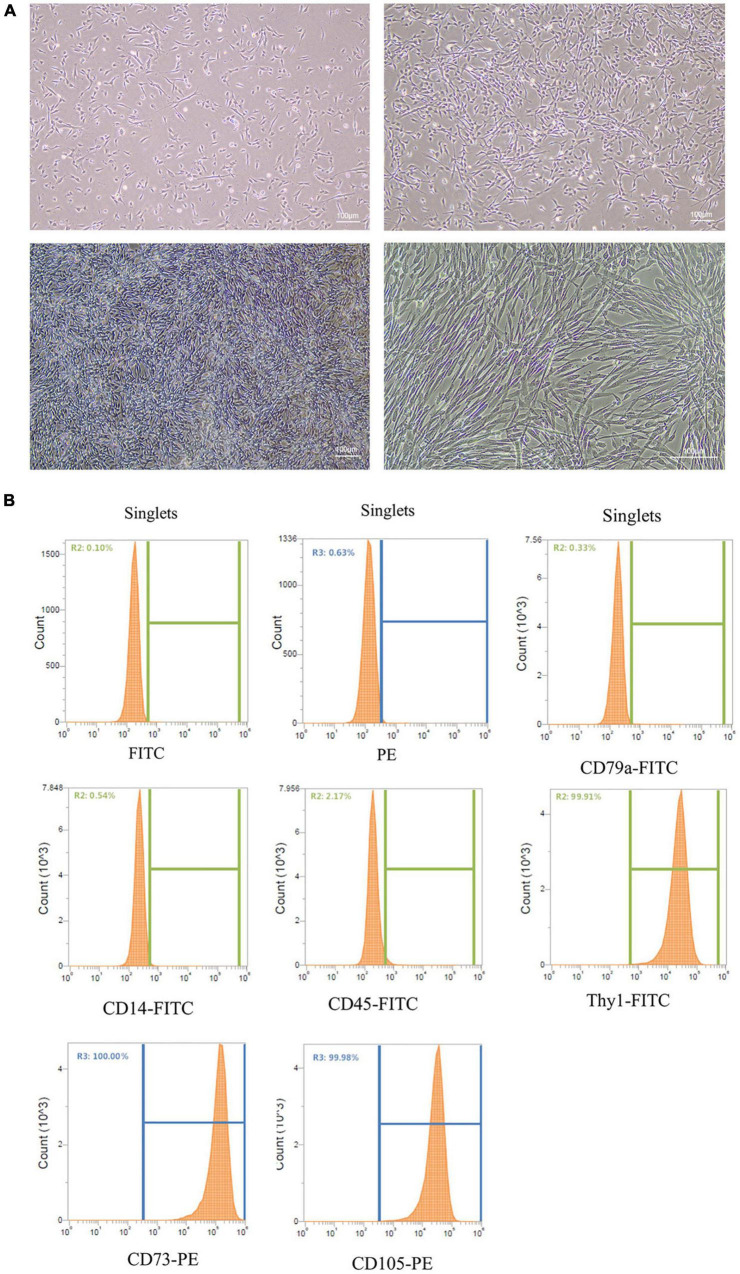
Culture and identification of UC-MSCs. **(A)** Culture of UC-MSCs. Scale bar = 100 μm. **(B)** Flow cytometry analysis showed that UC-MSCs were positive for CD105, Thy1 and CD73, but negative for CD79a, CD45 and CD14.

Stem cells have been used to treat skin damage, and stem cell-derived exosomes also have skin repair effects. Cell morphology can directly reflect the physiological state of the cells. After inoculation, the morphology of UC-MSCs incubated in serum-free medium showed a typical spindle shape (Figure 1A), with a uniform shape, clear outline, and ideal cell adhesion effect. Then, UC-MSCs surface markers were analyzed, and the expression of the cell surface antigens CD79a, CD45, CD14, CD105, Thy1, and CD73 on UC-MSCs was determined by flow cytometry. The results showed that CD105, Thy1, and CD73 expression was positive, and CD79a, CD45, and CD14 expression was negative (see Figure 1B). Through preliminary experiments, stable MSCs were obtained.

**FIGURE 2:**
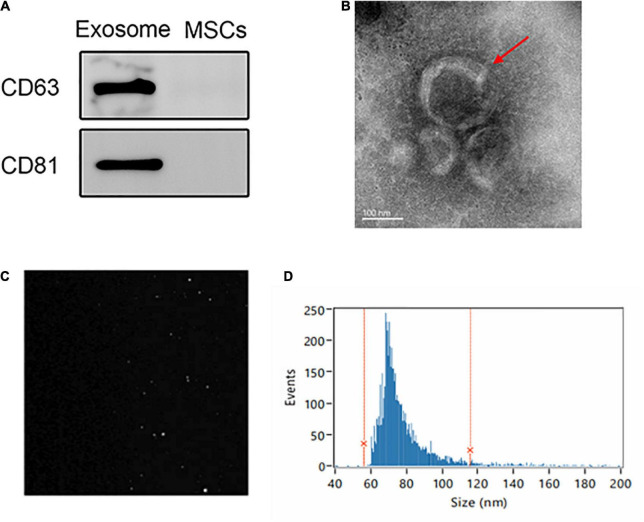
Extraction and identification of UC-MSC-Exo. **(A)** Expression of exosome markers (CD63 and CD81) examined by western blot analysis. **(B)** Representative images showing the morphology of UC-MSC-Exo by transmission electron microscopy. Scale bar = 100 nm. **(C)** The Brownian movement of the UC-MSC-Exo. **(D)** NTA analysis demonstrating the diameter of exosomes which ranged from 56.07 to 115.71 nm, with a mean diameter of 75.66 nm.

We isolated MSC-Exo from the supernatants of UC-MSCs and analyzed them *via* TEM, particle size measurement, and western blot assays. The exosome markers CD9 and TSG101 were detected in exosome samples by western blot assays (Figure 2A). TEM showed that the UC-MSC-Exo had a typical cup-shaped morphology of MSC-Exo (Figure 2B). Samples of the UC-MSC-Exo were analyzed with a NanoSight LM 10 system, the picture presents particles moving under Brownian motion (Figure 2C), and 95.94% of the particles were within the size range of 56.07–115.71 nm (Figure 2D). The cup-shaped morphology and size distribution of isolated samples corresponded to the TEM images. Together, these findings confirmed that MSC-Exo were successfully isolated, and that the UC-MSC-Exo in this study were similar to exosomes from other cell sources with respect to their morphology and properties.

### Umbilical Cord Mesenchymal Stem Cells-Exo Promoted the Proliferation of Skin Fibroblasts

**FIGURE 3:**
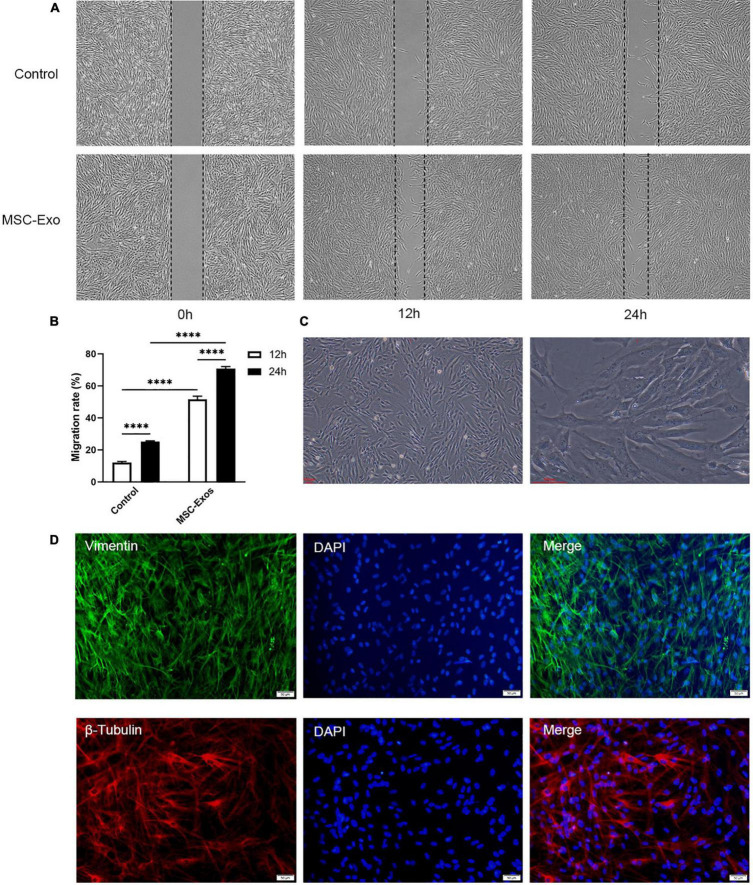
UC-MSC-Exo promote the growth of skin fibroblasts. **(A)** Scratch wound assay for HDFs treated with UC-MSC-Exo in three time points. **(B)** Statistical analysis of migration rates. ****p < 0.0001. Error bars indicate SDs of triplicate samples in a single representative experiment. SD, standard deviation. **(C)** The morphology of HDFs. Scale bar = 100 μm. **(D)** The immunofluorescence images of vimentin and β-tubulin. HDFs were immunolabeled for vimentin (green) and β-tubulin (red), and nucleic acid was signed with DAPI (blue). Scale bar = 50 μm.

To explore the ability of these exosomes to modulate fibroblast migration, we further conducted a scratch assay using HDFs to evaluate the wound healing capacity of the MSC-Exo. As shown in Figures 3A,B, the MSC-Exo group showed greater cell migration than the control group, as confirmed by optical microscopy. The distance of the migrated cells and the number of HDFs cocultured with MSC-Exo in the scratch area were significantly higher than those incubated in normal culture media (non-treated condition), with a statistically significant difference (*P* < 0.05). Fibroblasts are spindle-shaped or flattened star-shaped (Figure 3C), with vigorous functions and obvious protein synthesis and secretion activities. Vimentin is a marker protein of skin fibroblasts. β-Tubulin is the basic structural unit of intracellular microtubules and plays an indispensable role in maintaining cell shape, movement, and intracellular material transport. In fibroblasts, both vimentin (green), and β-tubulin (red) were well expressed (Figure 3D). Exosomes promote fibroblast proliferation and may have a positive effect on skin wound repair (Zhu et al., 2019).

### Umbilical Cord Mesenchymal Stem Cells-Exo Promoted the Secretion of Nerve Growth Factors by Skin Fibroblasts During Healing

**FIGURE 4:**
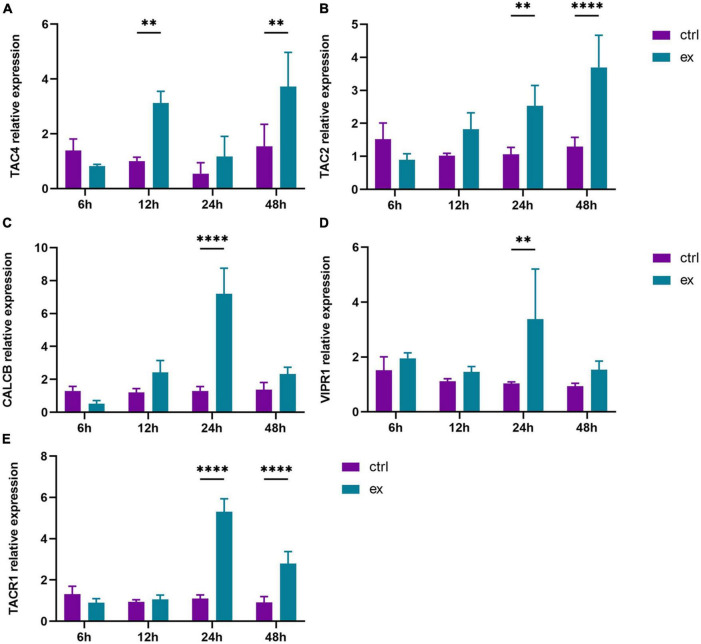
UC-MSC-Exo promote skin fibroblasts to secrete neural factors. The mRNA expression levels of TAC4 **(A)**, TAC2 **(B)**, CALCB **(C)**, VIPR1 **(D)**, TACR1 **(E)**, which were quantified by quantitative real-time RT-PCR. **p < 0.01, ****p < 0.0001. Error bars indicate SDs of triplicate samples in a single representative experiment.

To further define the relationship between exosomes and nerves, we investigated the effect of MSC-Exo on the secretion of nerve growth factors involved in the wound healing process. The level of nerve factors was determined by the expression of related mRNAs. In the control group, the expression of nerve growth factors was lower. Under MSC-Exo treatment, the content of nerve growth factors changed significantly, and the mRNA expression of TAC4 reached the highest level at 48 h (Figure 4A). The expression of TAC2 gradually increased with time, and compared with that in the control group, the mRNA expression of TAC2 was significantly increased at 24 and 48 h (Figure 4B). The expression of CALCB and VIPR1 reached the highest level at 24 h after treatment (Figures 4C,D). The highest expression level of TACR1 was found at 24 h (Figure 4E). The results indicated that MSC−Exo significantly promoted NGF secretion compared with the control, with a statistically significant difference (*P* < 0.05). This finding further verifies that exosomes may play a role in enhancing wound healing by promoting nerve repair.

### Umbilical Cord Mesenchymal Stem Cells-Exo Accelerated Cutaneous Wound Repair and Skin Regeneration During Wound Healing *in vivo*

**FIGURE 5:**
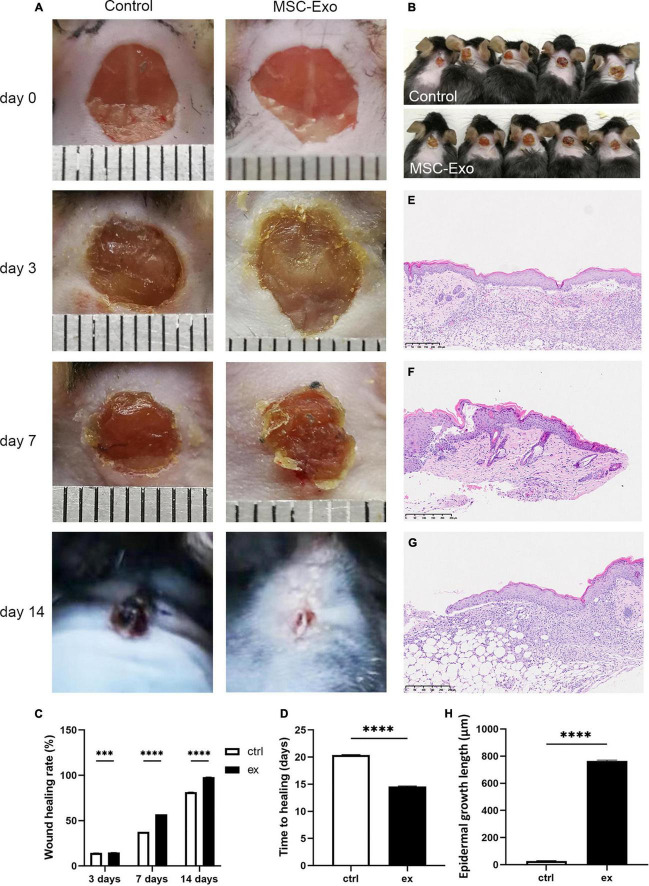
UC-MSC-Exo accelerates wound healing. **(A)** Representative images of the wound healing process in mice treated with control and MSC-Exo. **(B)** Wound healing situation in mice at 7 days. **(C)** Wound healing rate of experimental and control group. ***p < 0.0001, ****p < 0.0001. **(D)** Comparing of wound healing time after treatment with or without MSC-Exo. ****p < 0.0001. Histological features during healing of full-thickness skin wounds from normal mouse skin **(E)**, control mouse **(F)** and MSC-Exo mouse **(G)**. **(H)** Quantitative analysis of crawling distance after 14 days post-wounding. ****p < 0.0001, compared with the control group. Error bars indicate SDs of triplicate samples in a single representative experiment.

To verify the experimental results *in vitro*, we carried out *in vivo* experiments. To determine the effects of UC-MSC-Exo on wound healing and their role in this process, we generated full-thickness cutaneous wounds on the backs of each mouse. Full-thickness skin wounds on the backs of mice were treated with UC-MSC-Exo or PBS. The histological structure of the regenerated dermis was analyzed on day 14, including a 5 mm margin of intact skin, which was embedded in paraffin and serially sectioned from the border to the center point. Representative images of wound areas for each group on days 0, 3, 7, and 14 after wound treatment are shown in Figures 5A,B. The results of the *in vivo* experiment showed that the wounds in the control group were significantly red and swollen on the third day, and the inflammatory symptoms were stronger than those in the treatment group. There are 4 periods in the wound-healing process, namely, hemostasis, inflammatory response, proliferation, and remodeling. As shown in the figure, due to the effect of exosomes, the skin had passed the inflammatory phase, and the wound surface began to shrink. On the seventh day, the wounds in the control group showed some inflammatory reactions, while the experimental group showed scabbing. The wounds treated by UC-MSC-Exo recovered much more quickly compared with the control group (Figures 5C,D). HE staining showed that compared with the control group, the treatment group displayed enhanced growth of the epidermal tongue and a shortened repair time of the wound surface (Figures 5E–H), which was consistent with the *in vitro* results. These results demonstrate that MSC-Exo treatment accelerated wound healing.

### Umbilical Cord Mesenchymal Stem Cells-Exo Promoted Skin Nerve Fiber Regeneration

**FIGURE 6:**
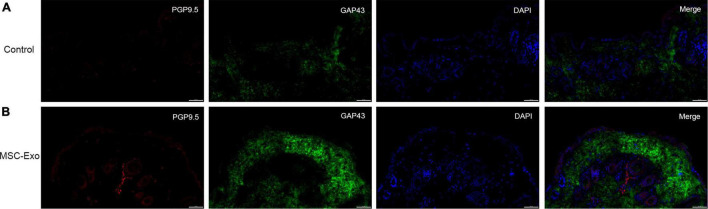
Nerve regeneration was analyzed by immunofluorescence staining. The full-thickness skin wounds of control group **(A)** and experimental group **(B)** were immunostained for PGP9.5 (red), GAP43 (green) and nucleic acid was signed with DAPI (blue). Scale bar = 50 μm.

**FIGURE 7:**
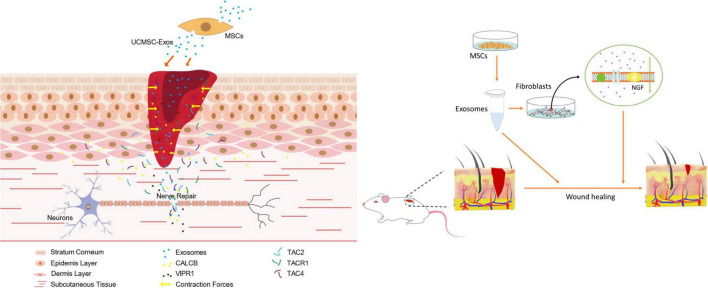
Schematic representation of UC-MSC-Exo promoting wound healing and nerve regeneration.

We performed systematic assessments of nerve regeneration by using immunofluorescence. Skin nerve fiber regeneration was analyzed by immunostaining for PGP9.5 and GAP43. As shown by immunofluorescence analysis (Figure 6), the skin nerve tissue specifically stained with an antibody against PGP9.5 exhibited an intense staining along the nerve fibers. PGP9.5 was found in skin fibers. In contrast, no such results were observed in the control groups, and control staining without antibody showed only background (Figures 6A,B). *In vitro*, UC-MSC-Exo promoted wound healing through cell proliferation and nerve damage repair. Skin cells are also involved in the process of wound repair and play a role through the expression of nerve-related markers and the regulation of nerve factors to promote nerve regeneration (Figure 7). This finding suggests that in the process of skin nerve injury, repairing nerves by improving the surrounding environment of skin nerves is a good strategy.

## Discussion

Wound healing is a complex process involving various cells, including keratinocytes, endothelial cells, and fibroblasts, as well as various cytokines (Singer and Clark, 1999; Broughton et al., 2006). In this study, we used UC-MSC-Exo to study the effect of stem cell exosomes on skin nerves during skin wound healing and applied them to a mouse skin injury model to verify their ability to promote skin nerve repair and regeneration. This treatment had a positive effect on damage repair. The main results of the study can be summarized as follows: (1) *In vitro*, UC-MSC-Exo stimulated fibroblast proliferation; (2) UC-MSC-Exo promoted the secretion of neural growth factors; and (3) UC-MSC-Exo accelerated wound healing in a full-thickness skin excision model. In this article, we showed evidence that UC-MSC-Exo are promising for the treatment of skin and nerve regeneration.

In recent years, biotechnology research on stem cell therapy and wound repair has gradually deepened (Tottoli et al., 2020). As an important part of stem cell paracrine signaling, exosomes play an important role in tissue regeneration. Exosomes are microvesicles, and many studies have shown that MSC-Exo have functions similar to those of MSCs, including repairing and regenerating tissues, inhibiting inflammatory responses, and regulating immunity (Ferreira and Gomes, 2018). In previous studies, a large number of studies have shown that stem cell exosomes have regeneration and repair effects on damaged skin, and MSCs from the umbilical cord, bone marrow, and adipose tissue are the most common sources (Peng et al., 2013; Chen et al., 2018; Wu et al., 2018), MSC-Exo have increasingly been explored as a valuable tool for mediating the healing of skin wounds (Hur et al., 2017; Guo et al., 2020). UC-MSCs have received extensive attention in the field of cell therapy and regenerative medicine and have been applied in different clinical fields (Mebarki et al., 2021). UC-MSC-Exo were also shown to effectively promote tissue repair and regeneration (Li et al., 2013; Zhang et al., 2015).

The human skin dermis includes multiple fibroblast subtypes, which are primarily responsible for the synthesis, deposition, and remodeling of dermal ECM, supporting the structural integrity of the skin, and are involved in the regulation of normal skin homeostasis, inflammation, and wound healing (Stunova and Vistejnova, 2018). Under the regulation of cytokines, fibroblasts proliferate, migrate, promote the synthesis and secretion of collagen and elastic fibers (Eming et al., 2017; Rodrigues et al., 2019), participate in the process of granulation tissue formation, wound contraction, scar formation, and tissue reconstruction (Reinke and Sorg, 2012; Driskell et al., 2013; Lynch and Watt, 2018), play an important role in the process of wound healing, and contribute to diverse healing outcomes, including non-healing chronic wounds or excessive scarring, such as hypertrophic scars (HTSs) and keloids (Meilang et al., 2022). Fibroblasts release signaling molecules in an autocrine or paracrine manner and mediate communication between surrounding cells, such as keratinocytes, endothelial cells, and macrophages, and participate in the process of wound healing together (Driskell and Watt, 2015; Lynch and Watt, 2018). In the proliferative period, fibroblasts greatly proliferate, migrate, and form granulation tissue and are mainly involved in resisting infection and replenishing wounds (Rognoni et al., 2018). Moreover, keratinocytes around the wound are induced to migrate to the wound to form new epidermal tissue. In the remodeling phase, myofibroblasts, endothelial cells, and macrophages differentiate from fibroblasts. Myofibroblasts synthesize and deposit ECM components that are the main source, generating strong contraction forces and bringing together the edges of the open wound, eventually forming collagen-rich scars (Rodero and Khosrotehrani, 2010; Meilang et al., 2022). Furthermore, recent studies have shown that the origin and phenotype of fibroblasts are important factors influencing the outcome of dermal repair, and dermal fibroblast heterogeneity can make wounds heal faster, with fewer scars, and has great potential for cell therapy (Meilang et al., 2022). Accelerating wound repair of the skin is of great clinical significance, both in difficult wound disease and in postoperative repair (Jinnin, 2010).

Orchestrated skin development can be achieved through data exchange and interference between intracellular and intercellular structures of the ectoderm and mesoderm (Itin, 2014). This complex process requires a highly coordinated interaction of several genetic signaling pathways (Deshmukh and Prashanth, 2012). The epidermis of the skin and its accessory structures develop from the ectoderm, which is also a key initiating participant in embryogenesis of the peripheral nervous system. Skin nerve repair and regeneration occur in two ways, namely, local stem cell proliferation and differentiation and the extension of healthy axons. Skin-derived precursor cells (SKPs) are pluripotent adult stem cells found in the dermis of human skin with good potential for multidirectional differentiation (Weng et al., 2020). They can proliferate and differentiate into nerve cells and glial cells *in vitro* and can effectively induce regeneration of skin sensory nerves (Toma et al., 2005; McKenzie et al., 2006). SKPs are involved in wound repair, and the possible mechanism is that in addition to directly participating in epidermal and dermal reconstruction, they may also participate in the nerve repair of wounds, suggesting that SKPs may become seed cells for the treatment of nervous system damage.

In the skin, there is a sophisticated network connecting cutaneous nerves and the local neuroendocrine and immune systems. The skin’s function and ability to respond to external stressors are regulated by the neuroendocrine system, including the regulated and coordinated production of neuropeptides (especially opioid peptides), neurohormones, neurotransmitters, and hormones, including steroids and secondary steroids. Skin cells have their own neuroendocrine network, with specific and well-functioning feedback regulatory circuits (Zmijewski and Slominski, 2011; Zmijewski et al., 2012). The neuroendocrine system has receptors for the expression of a variety of cytokines (Table 2), and cytokines act as immunotransmitters to exert their effects on the neuroendocrine system through their receptors. These factors induce/stimulate downstream signaling by activating the corresponding receptors. Many important signaling pathways are activated during wound healing and also play a role in embryonic skin development. The Wnt pathway is an important regulatory signaling pathway in growth and development. Research shows that Wnt signaling can be involved in early embryonic neural induction through interaction with bone morphogenetic proteins (BMPs) and FGF genes (Muñoz-Sanjuán and Brivanlou, 2002). During wound healing, Wnt signaling is activated by the wound and is involved in every subsequent stage of the healing process (Houschyar et al., 2020). Skin wounds express a variety of Wnt proteins in the early stage of healing, and the Wnt signaling pathway can regulate different proteins at different stages of wound repair. During the hemostasis and inflammation phases, local Wnt signaling begins to increase. Inflammatory cells release proinflammatory cytokines, growth factors, and vascular endothelial growth factors, increase vascular permeability, and promote fibroblast activity (Li et al., 2007). During the proliferative phase, β-catenin levels and transcriptional activity are elevated and become important regulators of fibroblast behavior in the proliferative phase of skin wound repair (Poon et al., 2009). During the proliferative phase, Wnt can activate stem cells, induce their self-renewal and proliferation, and stimulate active wound repair. Meanwhile, Wnt also plays an important role in angiogenesis, and endogenous enhancement of Wnt can correct vascular defects (Birdsey et al., 2015).

**TABLE 2:** The role of growth factors in cutaneous wound healing.

Growth factors	Main function	References
EGF	Re-epithelialization Enhances the production of fibronectin	Jiang et al., 1993; Yamamoto et al., 2013
TGF-α	Induces angiogenesis	Schaffer and Nanney, 1996
TGF-β	Inflammation Granulation tissue formation Re-epithelialization Matrix formation and remodeling	Eppley et al., 2004; Barrientos et al., 2008; Varkey et al., 2015
VEGF	Granulation tissue formation Stimulates (lymph) angiogenesis Enhances endothelial cell migration and proliferation	Morbidelli et al., 1996; Losi et al., 2013
PDGF	Granulation tissue formation Re-epithelialization Matrix formation and remodeling Activates macrophages to release growth factors	Barrientos et al., 2008; Borena et al., 2015
bFGF	Re-epithelialization Acts as a mitogen for fibroblasts Induces angiogenesis	Zhang et al., 2006; Inoue et al., 2009; Park et al., 2017
IGF	Re-epithelialization Stimulates fibroblast proliferation	Provenzano et al., 2007; Park et al., 2017

Neurotrophic factors are important bioactive molecules that regulate the proliferation of non-neuronal cells, as well as sensory, nerve axon sprouting/growth after injury (Terenghi, 1999), including NGFs, brain-derived neurotrophic factors (BDNFs), and glial cell-derived neurotrophic factors (GDNFs) (Madduri et al., 2009; Fadia et al., 2020; Table 3). NGF is a neurotrophic factor that is innervated by sensory and sympathetic neuronal projections, is responsible for establishing sensory innervation of the skin during development and maintaining the skin in adulthood (Indo, 2010), and can stimulate neurite outgrowth and nerve survival after injury (Hu et al., 2016; Zhao et al., 2016; Önger et al., 2017). Moreover, the regeneration of wound micro vessels is also related to innervation. The integrity of blood vessels is crucial to maintaining the homeostasis of the nervous system microenvironment, which is complementary to the regeneration and development of nerves. Neurotrophic factors can induce various effects on endothelial cells through autocrine and/or paracrine mechanisms (Nico et al., 2008; Gostynska et al., 2020). Studies have shown that NGF plays a role in angiogenesis, promotes the synthesis and secretion of VEGF (Ahluwalia et al., 2018), and can activate the PI3K/Akt and ERK/MAPK signaling pathways and downstream mTOR to mediate various NGF effects (Wang et al., 2016; Li et al., 2018). Topical administration of growth factors can improve wound healing (Graiani et al., 2004).

**TABLE 3:** The role of neurotrophic factors.

Neurotrophic factors	Main function	References
NGF	Nerve regeneration Regulator of neuronal differentiation Promote of neurite outgrowth and synaptic connection	Levi-Montalcini, 1987; Liu et al., 2022
BDNF	Re-epithelialization/keratinocyte proliferation Wound contraction Nerve regeneration	Marconi et al., 2003; Palazzo et al., 2012
GDNF	Schwann cell proliferation Nerve regeneration	Krakora et al., 2013; Itoyama et al., 2020
Neurotrophin-3 (NT-3)	Growth, proliferation, and maintenance of nerves Nerve regeneration	Liang et al., 1999
Substance P (SP)	Vasodilatation Polymorphonuclear cell infiltration Release of pro-inflammatory cytokines Re-epithelialization/keratinocyte proliferation Granulation tissue formation/fibroblast proliferation Angiogenesis Collagen maturation and remodeling Nerve regeneration	Liu et al., 2006; Ashrafi et al., 2016
Vasoactive intestinal peptide (VIP)	Vasodilatation Re-epithelialization/keratinocyte proliferation (−ve effect) Angiogenesis Collagen deposition Nerve regeneration	Rayan et al., 1995; Sternini, 1997
Cerebral dopamine neurotrophic factor (CDNF)	Nerve regeneration	Lindahl et al., 2017
Ciliary neurotrophic factor (CNTF)	Nerve regeneration Maturation, proliferation, and survival of OLGs Keep the maintenance of endoplasmic reticulum homeostasis	Kang et al., 2012
Mesencephalic astrocyte-derived neurotrophic factor (MANF)	Nerve regeneration Keep the maintenance of endoplasmic reticulum homeostasis	Lindahl et al., 2017

The progress of wound healing is complex, continuous, and dynamic. During the wound healing process, the wound site is constantly changing, including processes such as epithelialization and angiogenesis, in which various cells, cytokines, and growth factors work together, ultimately promoting wound healing. Cell proliferation is the key to tissue regeneration and repair, and the cell scratch assay is a common method used in the laboratory to analyze the ability of cells to migrate. Consistent with previous studies (Bakhtyar et al., 2018; Zhu et al., 2019), treatment with MSC-Exo significantly promoted the proliferation of fibroblasts on the experimental side (Figure 3). Nerves and various regulatory factors form a complex network of interactions (Chéret et al., 2014), and neurotrophic factors are considered a possible factor in skin wound healing. Subsequently, we detected the expression of neural factors in fibroblasts by polymerase chain reaction (Itoyama et al., 2020). To further test our hypothesis, we used animal models to explore the role of MSC-Exo. Then, we carried out an animal experiment with our materials to investigate wound repair from the appearance, nerve, and healing rates. Evidence obtained from mouse trauma models suggests that MSC-Exo could promote nerve fiber regeneration, which promotes skin wound healing. Skin regeneration repair and scar healing both have the nerve itself to repair, which is a crucial stage in the wound-healing process. Exosomes upregulate neurotrophic factor expression to enhance nerve regeneration, and stem cells and their exosomes show therapeutic advantages for neurological diseases (Kubiak et al., 2019; Saffari et al., 2021).

Physiological indicators in many *in vivo* experiments are used to assess wound healing. In our research, the MSC-Exo group exhibited greater regeneration of the epithelium and dermis, as well as a faster healing rate (Figure 5A), as evidenced by HE staining (Figure 5G). Moreover, many markers have and continue to be evaluated for diagnostic utility and are even beginning to be studied for prognostic utility. The neuronal biomarker PGP9.5 has been regarded as the most accurate for the visualization of epidermal nerves (Wang et al., 1990). GAP43 is a membrane protein that is involved in the process of nerve regeneration and is also a marker of nerve fiber regeneration (Fantini and Johansson, 1992; Denny, 2006). Immunohistochemical analysis of wound sites was performed to assess reinnervation using antibodies against the panaxonal marker (PGP9.5) and axonal regenerative marker (GAP43). PGP9.5 and GAP43 are appropriate markers for nerve regeneration studies. Our research results show that skin tissue was strongly GAP43/PGP9.5-positive after exosomes treatment (Figure 6), while nerve fibers were visualized primarily with PGP9.5, which is consistent with previous research results. These results suggest that MSC-Exo play an important role in the wound-healing process by repairing skin nerve fibers.

Large-scale skin injuries often lead to damage to subcutaneous nerve fibers, nerve endings, and receptors (Daly et al., 2012; Pateman et al., 2015). In the repair process, nerve scarring, or excessive extraneural fibrosis can affect nerve gliding, cause nerve tethering or compression, and decrease normal nerve conduction velocities, effectively limiting optimal functional recovery (Atkins et al., 2006; Wang M. L. et al., 2019). The disorganized conformation of fibroblasts is expressed in keloid scars and fibrosis and impedes regeneration at sites of nerve repair (Atkins et al., 2006). MSC-Exo provide new ideas for wound healing promotion and HTS prevention and are rich in protein, messenger RNA, and miRNAs as signaling molecules that inhibit collagen expression and myofibroblast proliferation and even remodel the ECM (Zhu et al., 2019).

Nerve repair is an important aspect of tissue repair that provides an explanation for the slow repair of skin tissue in many diseases, such as chronic skin wounds in diabetic neuropathy (Han et al., 2016). In diabetic foot ulcer (DFU), the main pathogenesis is related to peripheral neuropathy or peripheral nerve blood vessel damage caused by peripheral neuropathy, resulting in bone and joint damage in the patient’s foot, causing ulcers and infections, etc., in which a concomitant alteration of the nerve ending translates into a skin lesion, and makes its healing complex (Everett and Mathioudakis, 2018). The mechanisms underlying poor wound healing in diabetes are not fully understood. At present, there are various therapeutic strategies for DFU, and MSC-Exo exhibit higher proangiogenic and growth factor secretion activities, showing great application potential in clinical trials. MSC-Exo have achieved remarkable curative effects in a variety of diseases and have become an ideal solution for cell-free therapy in the field of regenerative medicine. In our study, we found that UC-MSC-Exo accelerated wound healing and exhibit neuroprotective and regenerative potential. Based on these findings, UC-MSC-Exo may show great therapeutic potential in tissue repair/regeneration. We need to further study the role of MSC-Exo in the surrounding microenvironment to reveal their mechanism in promoting nerve regeneration. In the future, the efficacy, safety, and potential risks of MSC-Exo in different diseases need further research and evaluation.

## Conclusion

Taken together, our findings presented in this study suggest that UC-MSC-Exo can produce therapeutic effects by promoting skin and nerve regeneration, which may be realized in clinical applications. However, further understanding of its molecular mechanism is necessary, and further exploration of the field of exosome biology is needed, which will ultimately contribute to the clinical application of exosomes in neural repair.

## Data Availability Statement

The original contributions presented in this study are included in the article/supplementary material, further inquiries can be directed to the corresponding authors.

## Ethics Statement

The animal study was reviewed and approved by the Animal Ethical and Welfare Committee (AEWC).

## Author Contributions

ZZ contributed to the conception of the study. MW, XZ, and HH contributed to the inception and experimental design and performed the experiments. XZ, HX, and JS performed the data collection and analyses and wrote the manuscript. QH and ZZ helped perform the analysis with constructive discussions. All authors contributed to this article and approved the submitted version.

## Conflict of Interest

The authors declare that the research was conducted in the absence of any commercial or financial relationships that could be construed as a potential conflict of interest.

## Publisher’s Note

All claims expressed in this article are solely those of the authors and do not necessarily represent those of their affiliated organizations, or those of the publisher, the editors and the reviewers. Any product that may be evaluated in this article, or claim that may be made by its manufacturer, is not guaranteed or endorsed by the publisher.

## References

[B1] Aheget H. Tristán-Manzano M. Mazini L. Cortijo-Gutierrez M. Benabdellah K. (2020). Exosome: a New Player in Translational Nanomedicine. *J. Clin. Med.* 9:2380. 10.3390/jcm9082380 32722531 PMC7463834

[B2] Ahluwalia A. Jones M. K. Hoa N. Zhu E. Brzozowski T. Tarnawski A. S. (2018). Reduced NGF in Gastric Endothelial Cells Is One of the Main Causes of Impaired Angiogenesis in Aging Gastric Mucosa. *Cell. Mol. Gastroenterol. Hepatol.* 6 199–213. 10.1016/j.jcmgh.2018.05.003 29992182 PMC6037903

[B3] Ashrafi M. Baguneid M. Bayat A. (2016). The Role of Neuromediators and Innervation in Cutaneous Wound Healing. *Acta Derm. Venereol.* 96 587–594. 10.2340/00015555-2321 26676806

[B4] Atkins S. Smith K. G. Loescher A. R. Boissonade F. M. O’Kane S. Ferguson M. W. (2006). Scarring impedes regeneration at sites of peripheral nerve repair. *Neuroreport* 17 1245–1249. 10.1097/01.wnr.0000230519.39456.ea 16951563

[B5] Bakhtyar N. Jeschke M. G. Herer E. Sheikholeslam M. Amini-Nik S. (2018). Exosomes from acellular Wharton’s jelly of the human umbilical cord promotes skin wound healing. *Stem Cell Res. Ther.* 9:193. 10.1186/s13287-018-0921-2 30005703 PMC6044104

[B6] Barrientos S. Stojadinovic O. Golinko M. S. Brem H. Tomic-Canic M. (2008). Growth factors and cytokines in wound healing. *Wound Repair Regen* 16 585–601. 10.1111/j.1524-475X.2008.00410.x 19128254

[B7] Birdsey G. M. Shah A. V. Dufton N. Reynolds L. E. Osuna Almagro L. Yang Y. (2015). The endothelial transcription factor ERG promotes vascular stability and growth through Wnt/β-catenin signaling. *Dev. Cell* 32 82–96. 10.1016/j.devcel.2014.11.016 25584796 PMC4292982

[B8] Borena B. M. Martens A. Broeckx S. Y. Meyer E. Chiers K. Duchateau L. (2015). Regenerative Skin Wound Healing in Mammals: State-of-the-Art on Growth Factor and Stem Cell Based Treatments. *Cell. Physiol. Biochem.* 36 1–23. 10.1159/000374049 25924569

[B9] Broughton G. Janis J. Attinger C. (2006). Wound Healing: An Overview. *Plast. Reconstr. Surg.* 117 1e–S–32e–S. 10.1097/01.prs.0000222562.60260.f9 16801750

[B10] Buckley G. Wong J. Metcalfe A. D. Ferguson M. (2012). Denervation affects regenerative responses in MRL/MpJ and repair in C57BL/6 ear wounds. *J. Anat.* 220 3–12. 10.1111/j.1469-7580.2011.01452.x 22066944 PMC3248659

[B11] Casado-Díaz A. Quesada-Gómez J. Dorado G. (2020). Extracellular Vesicles Derived From Mesenchymal Stem Cells (MSC) in Regenerative Medicine: applications in Skin Wound Healing. *Front. Bioeng. Biotechnol.* 8:146. 10.3389/fbioe.2020.00146 32195233 PMC7062641

[B12] Chen C. Y. Rao S. S. Ren L. Hu X. K. Tan Y. J. Hu Y. (2018). Exosomal DMBT1 from human urine-derived stem cells facilitates diabetic wound repair by promoting angiogenesis. *Theranostics* 8 1607–1623. 10.7150/thno.22958 29556344 PMC5858170

[B13] Chen R. Zhu Z. Ji S. Geng Z. Hou Q. Sun X. (2020). Sweat gland regeneration: current strategies and future opportunities. *Biomaterials* 255:120201. 10.1016/j.biomaterials.2020.120201 32592872

[B14] Chéret J. Lebonvallet N. Buhé V. Carre J. L. Misery L. Le Gall-Ianotto C. (2014). Influence of sensory neuropeptides on human cutaneous wound healing process. *J. Dermatol. Sci.* 74 193–203. 10.1016/j.jdermsci.2014.02.001 24630238

[B15] Ching R. C. Kingham P. J. (2015). The role of exosomes in peripheral nerve regeneration. *Neural Regen. Res.* 10 743–747. 10.4103/1673-5374.156968 26109947 PMC4468764

[B16] Daly W. Yao L. Zeugolis D. Windebank A. Pandit A. (2012). A biomaterials approach to peripheral nerve regeneration: bridging the peripheral nerve gap and enhancing functional recovery. *J. R. Soc. Interface* 9 202–221. 10.1098/rsif.2011.0438 22090283 PMC3243399

[B17] Denny J. B. (2006). Molecular mechanisms, biological actions, and neuropharmacology of the growth-associated protein GAP-43. *Curr. Neuropharmacol.* 4 293–304. 10.2174/157015906778520782 18654638 PMC2475799

[B18] Deshmukh S. Prashanth S. (2012). Ectodermal dysplasia: a genetic review. *Int. J. Clin. Pediatr. Dent.* 5 197–202. 10.5005/jp-journals-10005-1165 25206167 PMC4155886

[B19] Dong R. Liu Y. Yang Y. Wang H. Zhang Z. (2019). MSC-Derived Exosomes-Based Therapy for Peripheral Nerve Injury: A Novel Therapeutic Strategy. *BioMed Res. Int.* 2019:6458237. 10.1155/2019/6458237 31531362 PMC6719277

[B20] Dorschner R. A. Lee J. Cohen O. Costantini T. Eliceiri B. P. (2020). ECRG4 regulates neutrophil recruitment and CD44 expression during the inflammatory response to injury. *Sci. Adv.* 6:eaay0518. 10.1126/sciadv.aay0518 32195341 PMC7065879

[B21] Driskell R. R. Lichtenberger B. M. Hoste E. Kai K. Watt F. M. (2013). Distinct fibroblast lineages determine dermal architecture in skin development and repair. *Nature* 504 277–281. 10.1038/nature12783 24336287 PMC3868929

[B22] Driskell R. R. Watt F. M. (2015). Understanding fibroblast heterogeneity in the skin. *Trends Cell Biol.* 25 92–99. 25455110 10.1016/j.tcb.2014.10.001

[B23] Eming S. A. Wynn T. A. Martin P. (2017). Inflammation and metabolism in tissue repair and regeneration. *Science* 356 1026–1030. 10.1126/science.aam7928 28596335

[B24] Eppley B. L. Woodell J. E. Higgins J. (2004). Platelet quantification and growth factor analysis from platelet-rich plasma: implications for wound healing. *Plast. Reconstr. Surg.* 114 1502–1508. 10.1097/01.prs.0000138251.07040.51 15509939

[B25] Everett E. Mathioudakis N. (2018). Update on management of diabetic foot ulcers. *Ann. N. Y. Acad. Sci.* 1411 153–165. 10.1111/nyas.13569 29377202 PMC5793889

[B26] Fadia N. B. Bliley J. M. DiBernardo G. A. Crammond D. J. Schilling B. K. Sivak W. N. (2020). Long-gap peripheral nerve repair through sustained release of a neurotrophic factor in nonhuman primates. *Sci. Transl. Med.* 12:eaav7753. 10.1126/scitranslmed.aav7753 31969488

[B27] Falanga V. (2005). Wound healing and its impairment in the diabetic foot. *Lancet* 366 1736–1743. 16291068 10.1016/S0140-6736(05)67700-8

[B28] Fantini F. Johansson O. (1992). Expression of growth-associated protein 43 and nerve growth factor receptor in human skin: a comparative immunohistochemical investigation. *J. Invest. Dermatol.* 99 734–742. 10.1111/1523-1747.ep12614465 1281863

[B29] Farinazzo A. Turano E. Marconi S. Bistaffa E. Bazzoli E. Bonetti B. (2015). Murine adipose-derived mesenchymal stromal cell vesicles: invitro clues for neuroprotective and neuroregenerative approaches. *Cytotherapy* 17 571–578. 10.1016/j.jcyt.2015.01.005 25743633

[B30] Ferreira A. D. F. Gomes D. A. (2018). Stem Cell Extracellular Vesicles in Skin Repair. *Bioengineering* 6:4. 10.3390/bioengineering6010004 30598033 PMC6466099

[B31] Gonzalez A. C. D. O. Costa T. F. Andrade Z. D. A. Medrado A. R. A. P. (2016). Wound healing - A literature review. *An. Bras. Dermatol.* 91 614–620. 27828635 10.1590/abd1806-4841.20164741 PMC5087220

[B32] Gostynska N. Pannella M. Rocco M. L. Giardino L. Aloe L. Calzà L. (2020). The pleiotropic molecule NGF regulates the in vitro properties of fibroblasts, keratinocytes, and endothelial cells: implications for wound healing. *Am. J. Physiol. Cell Physiol.* 318 C360–C371. 10.1152/ajpcell.00180.2019 31774700

[B33] Graiani G. Emanueli C. Desortes E. Van Linthout S. Pinna A. Figueroa C. D. (2004). Nerve growth factor promotes reparative angiogenesis and inhibits endothelial apoptosis in cutaneous wounds of Type 1 diabetic mice. *Diabetologia* 47 1047–1054. 10.1007/s00125-004-1414-7 15164170

[B34] Guo S. Wang T. Zhang S. Chen P. Cao Z. Lian W. (2020). Adipose-derived stem cell-conditioned medium protects fibroblasts at different senescent degrees from UVB irradiation damages. *Mol. Cell Biochem.* 463 67–78. 10.1007/s11010-019-03630-8 31602539

[B35] Han J. W. Choi D. Lee M. Y. Huh Y. H. Yoon Y. S. (2016). Bone Marrow-Derived Mesenchymal Stem Cells Improve Diabetic Neuropathy by Direct Modulation of Both Angiogenesis and Myelination in Peripheral Nerves. *Cell Transplant.* 25 313–326. 10.3727/096368915x688209 25975801 PMC4889908

[B36] Houschyar K. S. Momeni A. Pyles M. N. Maan Z. N. Whittam A. J. Siemers F. (2015). Wnt signaling induces epithelial differentiation during cutaneous wound healing. *Organogenesis* 11 95–104. 10.1080/15476278.2015.1086052 26309090 PMC4879891

[B37] Houschyar K. S. Tapking C. Puladi B. Popp D. Duscher D. Rein S. (2020). Wnt signaling in cutaneous wound healing. *Handchir. Mikrochir. Plast. Chir.* 52 151–158. 10.1055/a-1017-3600 31724136

[B38] Hu J. Tian L. Prabhakaran M. P. Ding X. Ramakrishna S. (2016). Fabrication of Nerve Growth Factor Encapsulated Aligned Poly(ε-Caprolactone) Nanofibers and Their Assessment as a Potential Neural Tissue Engineering Scaffold. *Polymers* 8:54. 10.3390/polym8020054 30979150 PMC6432581

[B39] Hur W. Lee H. Y. Min H. S. Wufuer M. Lee C.-W. Hur J. A. (2017). Regeneration of full-thickness skin defects by differentiated adipose-derived stem cells into fibroblast-like cells by fibroblast-conditioned medium. *Stem Cell Res. Ther.* 8:92. 10.1186/s13287-017-0520-7 28427476 PMC5399413

[B40] Ibrahim A. E. Cheng K. Marbán E. (2014). Exosomes as critical agents of cardiac regeneration triggered by cell therapy. *Stem Cell Rep.* 2 606–619. 10.1016/j.stemcr.2014.04.006 24936449 PMC4050492

[B41] Indo Y. (2010). Nerve growth factor, pain, itch and inflammation: lessons from congenital insensitivity to pain with anhidrosis. *Expert Rev. Neurother.* 10 1707–1724. 10.1586/ern.10.154 20977328

[B42] Inoue S. Kijima H. Kidokoro M. Tanaka M. Suzuki Y. Motojuku M. (2009). The effectiveness of basic fibroblast growth factor in fibrin-based cultured skin substitute in vivo. *J. Burn Care Res.* 30 514–519. 10.1097/BCR.0b013e3181a28e4b 19349876

[B43] Itin P. H. (2014). Etiology and pathogenesis of ectodermal dysplasias. *Am. J. Med. Genet. A* 164a 2472–2477. 10.1002/ajmg.a.36550 24715647

[B44] Itoyama T. Yoshida S. Tomokiyo A. Hasegawa D. Hamano S. Sugii H. (2020). Possible function of GDNF and Schwann cells in wound healing of periodontal tissue. *J. Periodontal Res.* 55 830–839. 10.1111/jre.12774 32562261

[B45] Jiang C. K. Magnaldo T. Ohtsuki M. Freedberg I. M. Bernerd F. Blumenberg M. (1993). Epidermal growth factor and transforming growth factor alpha specifically induce the activation- and hyperproliferation-associated keratins 6 and 16. *Proc. Natl. Acad. Sci. U.S.A.* 90 6786–6790. 10.1073/pnas.90.14.6786 7688128 PMC47017

[B46] Jinnin M. (2010). Mechanisms of skin fibrosis in systemic sclerosis. *J. Dermatol.* 37 11–25. 10.1111/j.1346-8138.2009.00738.x 20175837

[B47] Kang S. S. Keasey M. P. Cai J. Hagg T. (2012). Loss of neuron-astroglial interaction rapidly induces protective CNTF expression after stroke in mice. *J. Neurosci.* 32 9277–9287. 10.1523/jneurosci.1746-12.2012 22764235 PMC3403837

[B48] Krakora D. Mulcrone P. Meyer M. Lewis C. Bernau K. Gowing G. (2013). Synergistic effects of GDNF and VEGF on lifespan and disease progression in a familial ALS rat model. *Mol. Ther.* 21 1602–1610. 10.1038/mt.2013.108 23712039 PMC3734670

[B49] Kubiak C. Grochmal J. Kung T. Cederna P. Midha R. Kemp S. (2019). Stem-cell Based Therapies to Enhance Peripheral Nerve Regeneration. *Muscle Nerve* 61 449–459. 10.1002/mus.26760 31725911

[B50] Lee Y. S. Sah S. K. Lee J. H. Seo K. W. Kang K. S. Kim T. Y. (2017). Human umbilical cord blood-derived mesenchymal stem cells ameliorate psoriasis-like skin inflammation in mice. *Biochem Biophys. Rep.* 9 281–288. 10.1016/j.bbrep.2016.10.002 28956015 PMC5614481

[B51] Levi-Montalcini R. (1987). The nerve growth factor: thirty-five years later. *Biosci. Rep.* 7 681–699. 10.1007/bf01116861 3322422

[B52] Li J. Chen J. Kirsner R. (2007). Pathophysiology of acute wound healing. *Clin. Dermatol.* 25 9–18. 10.1016/j.clindermatol.2006.09.007 17276196

[B53] Li T. Yan Y. Wang B. Qian H. Zhang X. Shen L. (2013). Exosomes derived from human umbilical cord mesenchymal stem cells alleviate liver fibrosis. *Stem Cells Dev.* 22 845–854. 10.1089/scd.2012.0395 23002959 PMC3585469

[B54] Li X. Li F. Li L. Li C. Zhong Y. (2018). Intranasal administration of nerve growth factor promotes angiogenesis via activation of PI3K/Akt signaling following cerebral infarction in rats. *Am. J. Transl. Res.* 10 3481–3492. 30662601 PMC6291726

[B55] Liang Y. Marcusson J. A. Johansson O. (1999). Light and electron microscopic immunohistochemical observations of p75 nerve growth factor receptor-immunoreactive dermal nerves in prurigo nodularis. *Arch. Dermatol. Res.* 291 14–21. 10.1007/s004030050378 10025723

[B56] Lindahl M. Saarma M. Lindholm P. (2017). Unconventional neurotrophic factors CDNF and MANF: structure, physiological functions and therapeutic potential. *Neurobiol. Dis.* 97 90–102. 10.1016/j.nbd.2016.07.009 27425895

[B57] Liu C. Y. Yin G. Sun Y. D. Lin Y. F. Xie Z. English A. W. (2020). Effect of exosomes from adipose-derived stem cells on the apoptosis of Schwann cells in peripheral nerve injury. *CNS Neurosci. Ther.* 26 189–196. 10.1111/cns.13187 31278850 PMC6978230

[B58] Liu J. Y. Hu J. H. Zhu Q. G. Li F. Q. Sun H. J. (2006). Substance P receptor expression in human skin keratinocytes and fibroblasts. *Br. J. Dermatol.* 155 657–662. 10.1111/j.1365-2133.2006.07408.x 16965412

[B59] Liu K. P. Ma W. Li C. Y. Li L. Y. (2022). Neurotrophic factors combined with stem cells in the treatment of sciatic nerve injury in rats: a meta-analysis. *Biosci. Rep.* 42:BSR20211399. 10.1042/bsr20211399 34897384 PMC8762346

[B60] Liu Z. Yu D. Xu J. Li X. Wang X. He Z. (2018). Human umbilical cord mesenchymal stem cells improve irradiation-induced skin ulcers healing of rat models. *Biomed. Pharmacother.* 101 729–736. 10.1016/j.biopha.2018.02.093 29524881

[B61] Losi P. Briganti E. Errico C. Lisella A. Sanguinetti E. Chiellini F. (2013). Fibrin-based scaffold incorporating VEGF- and bFGF-loaded nanoparticles stimulates wound healing in diabetic mice. *Acta Biomater.* 9 7814–7821. 10.1016/j.actbio.2013.04.019 23603001

[B62] Lynch M. Watt F. (2018). Fibroblast heterogeneity: implications for human disease. *J. Clin. Investig.* 128 26–35. 10.1172/JCI93555 29293096 PMC5749540

[B63] Madduri S. Papaloïzos M. Gander B. (2009). Synergistic effect of GDNF and NGF on axonal branching and elongation in vitro. *Neurosci. Res.* 65 88–97. 10.1016/j.neures.2009.06.003 19523996

[B64] Marconi A. Terracina M. Fila C. Franchi J. Bonté F. Romagnoli G. (2003). Expression and function of neurotrophins and their receptors in cultured human keratinocytes. *J. Invest. Dermatol.* 121 1515–1521. 10.1111/j.1523-1747.2003.12624.x 14675204

[B65] McKenzie I. A. Biernaskie J. Toma J. G. Midha R. Miller F. D. (2006). Skin-derived precursors generate myelinating Schwann cells for the injured and dysmyelinated nervous system. *J. Neurosci.* 26 6651–6660. 10.1523/jneurosci.1007-06.2006 16775154 PMC6674039

[B66] Mebarki M. Abadie C. Larghero J. Cras A. (2021). Human umbilical cord-derived mesenchymal stem/stromal cells: a promising candidate for the development of advanced therapy medicinal products. *Stem Cell Res. Ther.* 12:152. 10.1186/s13287-021-02222-y 33637125 PMC7907784

[B67] Meilang X. Ruilong Z. Lyn M. Christopher J. (2022). Dermal Fibroblast Heterogeneity and Its Contribution to the Skin Repair and Regeneration. *Adv. Wound Care* 11 87–107. 10.1089/wound.2020.1287 33607934

[B68] Morbidelli L. Chang C. H. Douglas J. G. Granger H. J. Ledda F. Ziche M. (1996). Nitric oxide mediates mitogenic effect of VEGF on coronary venular endothelium. *Am. J. Physiol.* 270 H411–H415. 10.1152/ajpheart.1996.270.1.H411 8769777

[B69] Muñoz-Sanjuán I. Brivanlou A. H. (2002). Neural induction, the default model and embryonic stem cells. *Nat. Rev. Neurosci.* 3 271–280. 10.1038/nrn786 11967557

[B70] Nico B. Mangieri D. Benagiano V. Crivellato E. Ribatti D. (2008). Nerve growth factor as an angiogenic factor. *Microvasc. Res.* 75 135–141. 10.1016/j.mvr.2007.07.004 17764704

[B71] Niimi Y. Fukuda S. Alharbi S. Prough D. S. Enkhbaatar P. (2020). Polyurethane foam for skin graft fixation in clinical-relevant ovine burn wound model for wound repair and regeneration research. *Regen. Ther.* 14 341–343. 10.1016/j.reth.2020.04.007 32490059 PMC7256436

[B72] Önger M. E. Delibaş B. Türkmen A. P. Erener E. Altunkaynak B. Z. Kaplan S. (2017). The role of growth factors in nerve regeneration. *Drug Discov. Ther.* 10 285–291. 10.5582/ddt.2016.01058 27746416

[B73] Oualla-Bachiri W. Fernández-González A. Quiones-Vico M. I. Arias-Santiago S. (2020). From Grafts to Human Bioengineered Vascularized Skin Substitutes. *Int. J. Mol. Sci.* 21:8197. 10.3390/ijms21218197 33147759 PMC7662999

[B74] Palazzo E. Marconi A. Truzzi F. Dallaglio K. Petrachi T. Humbert P. (2012). Role of neurotrophins on dermal fibroblast survival and differentiation. *J. Cell Physiol.* 227 1017–1025. 10.1002/jcp.22811 21503896

[B75] Park J. W. Hwang S. R. Yoon I. S. (2017). Advanced Growth Factor Delivery Systems in Wound Management and Skin Regeneration. *Molecules* 22:1259. 10.3390/molecules22081259 28749427 PMC6152378

[B76] Pateman C. J. Harding A. J. Glen A. Taylor C. S. Christmas C. R. Robinson P. P. (2015). Nerve guides manufactured from photocurable polymers to aid peripheral nerve repair. *Biomaterials* 49 77–89. 10.1016/j.biomaterials.2015.01.055 25725557

[B77] Peng L. H. Mao Z. Y. Qi X. T. Chen X. Li N. Tabata Y. (2013). Transplantation of bone-marrow-derived mesenchymal and epidermal stem cells contribute to wound healing with different regenerative features. *Cell Tissue Res.* 352 573–583. 10.1007/s00441-013-1609-7 23568655

[B78] Piipponen M. Li D. (2020). The Immune Functions of Keratinocytes in Skin Wound Healing. *Int. J. Mol. Sci.* 21:E8790. 10.3390/ijms21228790 33233704 PMC7699912

[B79] Poon R. Nik S. A. Ahn J. Slade L. Alman B. A. (2009). Beta-catenin and transforming growth factor beta have distinct roles regulating fibroblast cell motility and the induction of collagen lattice contraction. *BMC Cell Biol.* 10:38. 10.1186/1471-2121-10-38 19432963 PMC2691404

[B80] Pradhan L. Nabzdyk C. Andersen N. Logerfo F. Veves A. (2009). Inflammation and Neuropeptides: The Connection in Diabetic Wound Healing. *Expert Rev. Mol. Med.* 11:e2. 10.1017/S1462399409000945 19138453 PMC3708299

[B81] Provenzano P. P. Alejandro-Osorio A. L. Grorud K. W. Martinez D. A. Vailas A. C. Grindeland R. E. (2007). Systemic administration of IGF-I enhances healing in collagenous extracellular matrices: evaluation of loaded and unloaded ligaments. *BMC Physiol.* 7:2. 10.1186/1472-6793-7-2 17386107 PMC1851714

[B82] Rayan G. M. Johnson C. Pitha J. Cahill S. Said S. (1995). Vasoactive intestinal peptide and nerve growth factor effects on nerve regeneration. *J. Okla. State Med. Assoc.* 88 337–341. 7562142

[B83] Reinke J. M. Sorg H. (2012). Wound Repair and Regeneration. *Eur. Surg. Res.* 49 35–43. 22797712 10.1159/000339613

[B84] Robbins P. D. Morelli A. E. (2014). Regulation of immune responses by extracellular vesicles. *Nat. Rev. Immunol.* 14 195–208. 10.1038/nri3622 24566916 PMC4350779

[B85] Rodero M. Khosrotehrani K. (2010). Skin wound healing modulation by macrophages. *Int. J. Clin. Exp. Pathol.* 3 643–653. 20830235 PMC2933384

[B86] Rodrigues M. Kosaric N. Bonham C. A. Gurtner G. C. (2019). Wound Healing: A Cellular Perspective. *Physiol. Rev.* 99 665–706. 30475656 10.1152/physrev.00067.2017 PMC6442927

[B87] Rognoni E. Pisco A. O. Hiratsuka T. Sipilä K. H. Belmonte J. M. Mobasseri S. A. (2018). Fibroblast state switching orchestrates dermal maturation and wound healing. *Mol. Syst. Biol.* 14:e8174. 10.15252/msb.20178174 30158243 PMC6113774

[B88] Roh J. L. Lee J. Kim E. H. Shin D. (2017). Plasticity of oral mucosal cell sheets for accelerated and scarless skin wound healing. *Oral Oncol.* 75 81–88. 10.1016/j.oraloncology.2017.10.024 29224829

[B89] Saffari S. Saffari T. Ulrich D. Hovius S. Shin A. (2021). The interaction of stem cells and vascularity in peripheral nerve regeneration. *Neural Regen. Res.* 16 1510–1517. 10.4103/1673-5374.303009 33433464 PMC8323682

[B90] Schaffer C. J. Nanney L. B. (1996). Cell biology of wound healing. *Int. Rev. Cytol.* 169 151–181. 10.1016/s0074-7696(08)61986-5 8843654

[B91] Sen C. K. Gordillo G. M. Roy S. Kirsner R. Lambert L. Hunt T. K. (2009). Human skin wounds: a major and snowballing threat to public health and the economy. *Wound Repair Regen.* 17 763–771. 10.1111/j.1524-475x.2009.00543.x 19903300 PMC2810192

[B92] Shaw T. J. Martin P. (2009). Wound repair at a glance. *J. Cell Sci.* 122 3209–3213. 10.1242/jcs.031187 19726630 PMC2736861

[B93] Shiue S. J. Rau R. H. Shiue H. S. Hung Y. W. Li Z. X. Yang K. D. (2019). Mesenchymal stem cell exosomes as a cell-free therapy for nerve injury-induced pain in rats. *Pain* 160 210–223. 10.1097/j.pain.0000000000001395 30188455

[B94] Singer A. J. Clark R. A. F. (1999). Cutaneous Wound Healing. *N. Engl. J. Med.* 341 738–746. 10.1056/nejm199909023411006 10471461

[B95] Smith P. G. Liu M. (2002). Impaired cutaneous wound healing after sensory denervation in developing rats: effects on cell proliferation and apoptosis. *Cell Tissue Res.* 307 281–291. 10.1007/s00441-001-0477-8 11904764

[B96] Sternini C. (1997). Organization of the peripheral nervous system: autonomic and sensory ganglia. *J. Investig. Dermatol. Symp. Proc.* 2 1–7. 10.1038/jidsymp.1997.2 9487007

[B97] Stunova A. Vistejnova L. (2018). Dermal fibroblasts-A heterogeneous population with regulatory function in wound healing. *Cytokine Growth Factor Rev.* 39 137–150. 10.1016/j.cytogfr.2018.01.003 29395658

[B98] Su S.-A. Xie Y. Fu Z. Wang Y. Wang J.-A. Xiang M. (2017). Emerging role of exosome-mediated intercellular communication in vascular remodeling. *Oncotarget* 8 25700–25712. 10.18632/oncotarget.14878 28147325 PMC5421963

[B99] Terenghi G. (1999). Peripheral nerve regeneration and neurotrophic factors. *J. Anat.* 194 1–14. 10.1046/j.1469-7580.1999.19410001.x 10227662 PMC1467889

[B100] Toma J. G. McKenzie I. A. Bagli D. Miller F. D. (2005). Isolation and characterization of multipotent skin-derived precursors from human skin. *Stem Cells* 23 727–737. 10.1634/stemcells.2004-0134 15917469

[B101] Torre S. Fernández-González A. Quiones-Vico M. I. Montero-Vilchez T. Arias-Santiago S. (2020). Bioengineered Skin Intended as In Vitro Model for Pharmacosmetics, Skin Disease Study and Environmental Skin Impact Analysis. *Biomedicines* 8:464. 10.3390/biomedicines8110464 33142704 PMC7694072

[B102] Tottoli E. Dorati R. Genta I. Chiesa E. Pisani S. Conti B. (2020). Skin Wound Healing Process and New Emerging Technologies for Skin Wound Care and Regeneration. *Pharmaceutics* 12:735. 10.3390/pharmaceutics12080735 32764269 PMC7463929

[B103] Tsai S. C. Yang K. D. Chang K. H. Lin F. C. Chou R. H. Li M. C. (2021). Umbilical Cord Mesenchymal Stromal Cell-Derived Exosomes Rescue the Loss of Outer Hair Cells and Repair Cochlear Damage in Cisplatin-Injected Mice. *Int. J. Mol. Sci.* 22:6664. 10.3390/ijms22136664 34206364 PMC8267798

[B104] Varkey M. Ding J. Tredget E. E. (2015). Advances in Skin Substitutes-Potential of Tissue Engineered Skin for Facilitating Anti-Fibrotic Healing. *J. Funct. Biomater.* 6 547–563. 10.3390/jfb6030547 26184327 PMC4598670

[B105] Vesna B. Desiree V. Claas-Tido P. Sarah S. Vogt P. M. Christine R. (2018). Effect of Exosomes from Rat Adipose-Derived Mesenchymal Stem Cells on Neurite Outgrowth and Sciatic Nerve Regeneration After Crush Injury. *Mol. Neurobiol.* 56 1812–1824. 10.1007/s12035-018-1172-z 29931510 PMC6394792

[B106] Vu N. B. Nguyen H. T. Palumbo R. Pellicano R. Fagoonee S. Pham P. V. (2021). Stem cell-derived exosomes for wound healing: current status and promising directions. *Minerva Med.* 112 384–400. 10.23736/s0026-4806.20.07205-5 33263376

[B107] Wang J. He C. Zhou T. Huang Z. Zhou L. Liu X. (2016). NGF increases VEGF expression and promotes cell proliferation via ERK1/2 and AKT signaling in Müller cells. *Mol. Vis.* 22 254–263. 27081296 PMC4812506

[B108] Wang L. Hilliges M. Jernberg T. Wiegleb-Edström D. Johansson O. (1990). Protein gene product 9.5-immunoreactive nerve fibres and cells in human skin. *Cell Tissue Res.* 261 25–33. 10.1007/bf00329435 2143435

[B109] Wang M. Yuan Q. Xie L. (2018). Mesenchymal Stem Cell-Based Immunomodulation: properties and Clinical Application. *Stem Cells Int.* 2018:3057624. 10.1155/2018/3057624 30013600 PMC6022321

[B110] Wang M. L. Rivlin M. Graham J. G. Beredjiklian P. K. (2019). Peripheral nerve injury, scarring, and recovery. *Connect Tissue Res.* 60 3–9. 10.1080/03008207.2018.1489381 30187777

[B111] Wang X. Jiao Y. Pan Y. Zhang L. Gong H. Qi Y. (2019). Fetal Dermal Mesenchymal Stem Cell-Derived Exosomes Accelerate Cutaneous Wound Healing by Activating Notch Signaling. *Stem Cells Int.* 2019:2402916. 10.1155/2019/2402916 31281370 PMC6590601

[B112] Weng T. Wu P. Zhang W. Zheng Y. Li Q. Jin R. (2020). Regeneration of skin appendages and nerves: current status and further challenges. *J. Transl. Med.* 18:53. 10.1186/s12967-020-02248-5 32014004 PMC6996190

[B113] Whyte J. L. Smith A. A. Helms J. A. (2012). Wnt signaling and injury repair. *Cold Spring Harb. Perspect. Biol.* 4:a008078. 10.1101/cshperspect.a008078 22723493 PMC3405869

[B114] Woodby B. Penta K. Pecorelli A. Lila M. Valacchi G. (2020). Skin Health from the Inside Out. *Annu. Rev. Food Sci. Technol.* 11 235–254. 31905017 10.1146/annurev-food-032519-051722

[B115] Woodley D. T. (2017). Distinct Fibroblasts in the Papillary and Reticular Dermis: implications for Wound Healing. *Dermatol. Clin.* 35 95–100. 10.1016/j.det.2016.07.004 27890241

[B116] Wu P. Zhang B. Shi H. Qian H. Xu W. (2018). MSC-exosome: a novel cell-free therapy for cutaneous regeneration. *Cytotherapy* 20 291–301. 10.1016/j.jcyt.2017.11.002 29434006

[B117] Yamamoto A. Shimizu N. Kuroyanagi Y. (2013). Potential of wound dressing composed of hyaluronic acid containing epidermal growth factor to enhance cytokine production by fibroblasts. *J. Artif Organs* 16 489–494. 10.1007/s10047-013-0726-0 24013475

[B118] Ying Q. Zhang Q. Cai X. Fei L. Ma Z. (2017). Exosomes derived from miR-181-5p-modified adipose-derived mesenchymal stem cells prevent liver fibrosis via autophagy activation. *J. Cell. Mol. Med.* 21:2491. 10.1111/jcmm.13170 28382720 PMC5618698

[B119] Yu T. Gao M. Yang P. Liu D. Wang D. Song F. (2019). Insulin promotes macrophage phenotype transition through PI3K/Akt and PPAR-γ signaling during diabetic wound healing. *J. Cell. Physiol.* 234 4217–4231. 10.1002/jcp.27185 30132863

[B120] Zhang B. Wu X. Zhang X. Sun Y. Yan Y. Shi H. (2015). Human umbilical cord mesenchymal stem cell exosomes enhance angiogenesis through the Wnt4/β-catenin pathway. *Stem Cells Transl. Med.* 4 513–522. 10.5966/sctm.2014-0267 25824139 PMC4414225

[B121] Zhang X. Ibrahimi O. A. Olsen S. K. Umemori H. Mohammadi M. Ornitz D. M. (2006). Receptor specificity of the fibroblast growth factor family. The complete mammalian FGF family. *J. Biol. Chem.* 281 15694–15700. 10.1074/jbc.M601252200 16597617 PMC2080618

[B122] Zhao J. Ding Y. He R. Huang K. Liu L. Jiang C. (2020). Dose-effect relationship and molecular mechanism by which BMSC-derived exosomes promote peripheral nerve regeneration after crush injury. *Stem Cell Res. Ther.* 11:360. 10.1186/s13287-020-01872-8 32811548 PMC7437056

[B123] Zhao Y. Z. Jiang X. Xiao J. Lin Q. Yu W. Z. Tian F. R. (2016). Using NGF heparin-poloxamer thermosensitive hydrogels to enhance the nerve regeneration for spinal cord injury. *Acta Biomater.* 29 71–80. 10.1016/j.actbio.2015.10.014 26472614 PMC7517710

[B124] Zhu Z. Hou Q. Li M. Fu X. (2019). Molecular mechanism of myofibroblast formation and strategies for clinical drugs treatments in hypertrophic scars. *J. Cell. Physiol.* 235 4109–4119. 10.1002/jcp.29302 31612497

[B125] Zmijewski M. Skobowiat C. Zbytek B. Slominski R. Steketee J. (2012). Sensing the environment: regulation of local and global homeostasis by the skin neuroendocrine system. *Adv. Anat. Embryol. Cell Biol.* 212 v–115. 10.1007/978-3-642-19683-6_1 22894052 PMC3422784

[B126] Zmijewski M. A. Slominski A. T. (2011). Neuroendocrinology of the skin. *Derm.-Endocrinol.* 3 3–10. 10.4161/derm.3.1.14617 21519402 PMC3051846

